# Supplementary figures and images for: Carrot Pomace Polysaccharide (CPP) Improves Influenza Vaccine Efficacy in Immunosuppressed Mice via Dendritic Cell Activation
Source: Nutrients. 2020 Sep 9;12(9):2740. doi: 10.3390/nu12092740 (PMC7551730; doi:10.3390/nu12092740)

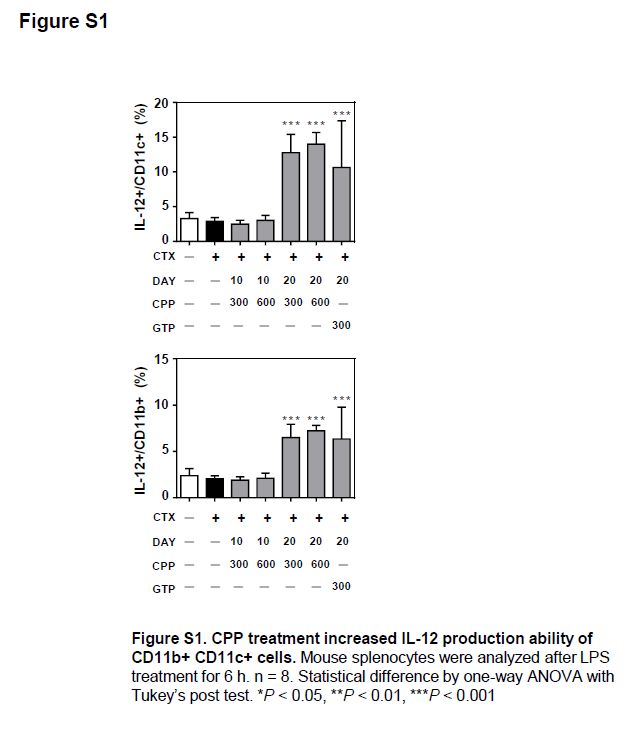

Supplement: Supplementary file 1 [file nutrients-12-02740-s001.zip › nutrients-898665-supplementary.PNG]
